# M2 macrophage infiltration drives tumor progression and identifies a multigene prognostic signature in esophageal cancer

**DOI:** 10.3389/fimmu.2025.1659048

**Published:** 2026-02-02

**Authors:** Guangxia Wei, Chunlin Ye, Yunhe Huang, Zan Luo, Bin Xu, Junyu Li

**Affiliations:** 1 Department of Thoracic Surgery, Nanfang Hospital, Southern Medical University, Guangzhou, China; 2 Department of Thoracic Surgery, the First Affiliated Hospital, Jiangxi Medical College, Nanchang University, Nanchang, China; 3 Department of Radiation Oncology, Jiangxi Key Laboratory of Oncology, Jiangxi Cancer Hospital (The Second Affiliated Hospital of Nanchang Medical College), Nanchang, China

**Keywords:** biomarkers, esophageal cancer, M2 tumor-associated macrophages (M2 TAMs), microenvironment (TIME), prognostic model, single-cell RNA sequencing (scRNA-seq), tumor immune

## Abstract

**Objective:**

This study aimed to elucidate the mechanistic role of M2 tumor-associated macrophages in esophageal cancer (EC) progression and to construct an M2 macrophage–related gene signature for prognostic prediction.

**Methods:**

Integrated analyses of single-cell RNA sequencing (scRNA-seq) and bulk RNA-seq data of EC were performed. scRNA-seq data were processed with Seurat and annotated using SingleR. Immune infiltration was evaluated through ssGSEA and CIBERSORT. Weighted gene coexpression network analysis (WGCNA) was used to identify M2 macrophage–associated modules, and candidate genes were intersected with differentially expressed genes (DEGs). Functional enrichment analyses were performed, and a prognostic risk model was established through multivariate Cox regression analysis. The functional roles of key genes were validated through *in vitro* and *in vivo* experiments.

**Results:**

Twelve cell types were identified by scRNA-seq, with macrophages representing the predominant immune population. M2 macrophages formed the major immunosuppressive subtype and were negatively associated with patient survival. WGCNA and DEG analysis identified 25 M2-related genes, from which a four-gene prognostic signature (*SPINK5*, *A2ML1*, *IL1RN*, *IL36G*) was constructed. The model effectively stratified EC patients into distinct risk groups with significantly different survival outcomes. Further *in vitro* experiments demonstrated that silencing *IL1RN* and *IL36G* in macrophages markedly suppressed the malignant phenotypes of esophageal cancer cells. Complementary *in vivo* experiments provided additional evidence, further indicating that IL1RN and IL36G play important functional roles in overall tumor progression.

**Conclusion:**

A four-gene M2 macrophage–related prognostic model provides reliable prediction of clinical outcomes in EC. Among these genes, *IL1RN* and *IL36G* function as key regulators whose silencing inhibits M2 polarization and attenuates tumor proliferation, invasion, migration, and epithelial–mesenchymal transition.

## Introduction

Esophageal cancer (EC), which mainly includes esophageal squamous cell carcinoma (ESCC) and esophageal adenocarcinoma (EAC), represents one of the most prevalent malignancies of the digestive tract. It ranks among the top six causes of cancer-related mortality worldwide and occurs with particularly high frequency in Asia and Africa, where overall prognosis remains poor (1, 2). Major risk factors include tobacco use, alcohol consumption, metastatic potential, pre-existing esophageal dysfunction, and genetic susceptibility (3, 4). Current therapeutic options encompass gene therapy, chemotherapy, radiotherapy, esophagectomy, and combined chemoradiation strategies (5). Despite advances in clinical management, the overall survival of EC remains unsatisfactory, as reflected by persistently low 5-year survival rates (6).

The tumor microenvironment (TME), composed of diverse cellular and acellular components, is essential for tumor initiation and progression. Among these, tumor-associated macrophages (TAMs) have garnered considerable attention due to their strong association with poor clinical outcomes in multiple malignancies (7). Macrophages exhibit remarkable plasticity and can polarize into distinct phenotypes, including the pro-inflammatory M1 and anti-inflammatory M2 subtypes, in response to microenvironmental stimuli (8, 9). M2 TAMs have been shown to induce epithelial-mesenchymal transition in ESCC cells (10). In addition, serine protease inhibitor Kazal type-5 (*SPINK5*), identified as a novel tumor suppressor, is markedly downregulated during EC progression and correlates with poor differentiation and lymph node metastasis (11). Previous research has also identified duplicate variants of alpha-2 macroglobulin-like 1 (A2ML1) as important risk factors for otitis media in both the indigenous Filipino population and children in the United States (12). The tumor suppressive role of interleukin-1 receptor antagonist (IL1RN) has been demonstrated in ESCC (13). Evidence regarding the role of interleukin 36 gamma (IL36G) in EC remains limited. In this study, single-cell RNA sequencing (scRNA-seq) and bulk RNA-seq datasets of EC were obtained from public repositories and analyzed using multiple computational biology approaches. Integrated analyses of tumor single-cell profiles, transcriptomic data, and clinical information revealed substantial intratumoral heterogeneity in EC. After identifying M2 macrophages as strongly associated with patient prognosis, a four-gene prognostic signature comprising *SPINK5*, *A2ML1*, *IL1RN*, and *IL36G* was established to facilitate survival prediction and risk stratification in EC.

## Materials and methods

### Data acquisition

scRNA-seq data (GSE145370), comprising seven EC tumor samples, and microarray data (GSE77861), including paired normal and tumor tissues from seven patients with ESCC, were obtained from the Gene Expression Omnibus (GEO) database (http://www.ncbi.nlm.nih.gov/geo/). Bulk RNA-seq data and corresponding clinical information for EC (TCGA-ESCA; normal, n = 11; tumor, n = 162) and esophageal adenocarcinoma (TCGA-ESAD; normal, n = 10; tumor, n = 80) cohorts were retrieved from the UCSC Xena platform (https://xenabrowser.net/). As all datasets were derived from publicly available sources, no additional ethical approval or informed consent was required.

### scRNA-seq analysis

scRNA-seq data were processed using the Seurat package (14). Quality control procedures retained high-quality cells based on the following thresholds: nFeature_RNA greater than 200 to remove empty droplets and low-content cells, nCount_RNA between 1000 and 20000 to exclude low-expression cells and potential doublets, and percent.mt less than 20% to eliminate cells showing excessive mitochondrial gene expression indicative of cellular stress or apoptosis (15, 16). Correlations among nCount_RNA, percent.mt, and nFeature_RNA were assessed to evaluate data quality.

Gene expression matrices were normalized using the LogNormalize function. Principal component analysis (PCA) was applied for dimensionality reduction, and significant principal components (PCs) were identified using the JackStraw method, which iteratively resampled 1% of the expression matrix to generate a null distribution. JackStrawPlot was used to visualize the P-value distribution of the top 40 PCs, while ElbowPlot analysis was used to determine the optimal inflection point. Based on both approaches, the top 20 PCs were selected for downstream t-distributed stochastic neighbor embedding (tSNE) analysis, performed with a perplexity of 30 and a seed value of 100 to ensure reproducibility (17).

### Single-sample gene-set enrichment analysis

ssGSEA was performed using the “GSVA” package to quantify enrichment scores for functional marker genes associated with 22 immune cell types (18). The hallmark gene set used for analysis was obtained from **Supplementary Materials** of previously published studies (19, 20).

### Identification of key modules for M2 macrophage infiltration

The CIBERSORT algorithm was applied to estimate M2 macrophage infiltration in the TCGA-ESCA dataset with 1,000 permutations, retaining only samples with *p* < 0.05 (21). CIBERSORT, a deconvolution method based on normalized gene expression profiles, quantifies immune cell composition and has been validated by fluorescence-activated cell sorting (FACS). Gene expression matrices with standard annotations were uploaded to the CIBERSORT web portal (http://cibersort.stanford.edu/) using the LM22 signature matrix and 1,000 simulations (22). Weighted gene coexpression network analysis (WGCNA) was performed using the WGCNA package in R (23). The top 25% of genes ranked by standard deviation were selected to ensure an optimal balance between biological relevance and computational efficiency, following WGCNA recommendations and previous studies (24). The module preservation function was applied to evaluate the robustness of identified modules (25). A signed scale-free coexpression network was constructed using a soft threshold β of 4, which achieved a scale-free topology fit index (R^2^) of 0.90. The module exhibiting the strongest correlation with M2 macrophage infiltration was designated as the key M2-associated module, and its member genes were extracted for downstream analyses.

### Analysis of differential gene expression on microarray GSE77861

Differentially expressed genes (DEGs) in the GSE77861 microarray dataset were identified using the “limma” and “impute” packages in R. *P*-values were adjusted by the false discovery rate (FDR) method, with screening thresholds set at |logFC| > 1 and adjusted p < 0.05. The overlap between DEGs and WGCNA-derived module genes was visualized via an online Venn diagram tool (http://bioinformatics.psb.ugent.be/webtools/Venn/).

### Protein-protein interaction analysis

STRING database (https://string-db.org/) was used to construct a PPI network based on the intersecting genes, with the species parameter set to human. The resulting interaction data were imported into Cytoscape 3.6.0 for visualization. Degree value and Combined score were used to evaluate node importance, and the top 15 nodes were ranked according to Degree value (26).

### Gene function enrichment analysis

Functional enrichment analyses of candidate genes or module genes were conducted using the SangerBox online platform (http://sangerbox.com/Tool). Gene Ontology (GO) enrichment results were visualized using bubble plots for biological process (BP), cellular component (CC), and molecular function (MF) categories. Kyoto Encyclopedia of Genes and Genomes (KEGG) pathway enrichment was presented using bar plots, bubble charts, and circular diagrams to illustrate significantly enriched signaling pathways.

### Survival analysis and receiver operating characteristic curve construction

Survival analysis was performed using the “survival” package in R (https://CRAN.R-project.org/package=survival). Hazard ratios (HRs) for patients with EC were estimated using the Cox proportional hazards regression model. Predictive performance of the prognostic model was evaluated using Receiver operating characteristic (ROC) curves generated with the “pROC” package (https://cran.r-project.org/web/packages/pROC/index.html).

### Immunofluorescence staining

Tumor and matched adjacent tissues from patients with EC (n = 5 per group) were fixed in 4% paraformaldehyde (Sigma-Aldrich, USA) for 4 h, embedded in OCT (Tissue-Tek, Sakura Finetek, USA), and sectioned at 7 μm. Sections were permeabilized with 0.3% Triton X-100 and blocked with 5% BSA (Sigma-Aldrich, USA) for 1 h. Primary antibodies against IL1RN (ab303490, 1:200, Abcam, UK), IL36G (ab239526, 1:200, Abcam, UK), and CD163 (sc-20066, 1:200, Santa Cruz, USA) were incubated overnight at 4°C. After washing, Alexa Fluor 488– or 594–conjugated secondary antibodies (1:500, Invitrogen, USA) were applied for 1 h, followed by DAPI staining (1 μg/mL, Thermo Fisher Scientific, USA). Images were obtained with a Leica TCS SP8 confocal microscope (Germany), and fluorescence intensity was analyzed using ImageJ. Co-localization of IL1RN, IL36G, and CD163 was assessed to verify their association with M2 macrophages.

### Cell culture

The human monocyte cell line THP-1 (ATCC TIB-202) was cultured in 6-well plates and treated with 100 ng/mL phorbol 12-myristate 13-acetate (PMA) (Sigma-Aldrich, USA) for 24 h to induce differentiation into M0 macrophages. After replacing the PMA-containing medium with fresh complete medium, cells were incubated for another 24 h.

Human EC cell line EC109 (CBP60496) and TE-1 (CBP60655) (Nanjing COBIOER Biosciences, China), and the normal esophageal epithelial cell line ET-1A (CRL-2692, ATCC), were maintained in RPMI-1640 medium enriched with 10% fetal bovine serum (FBS, Gibco) and 1% penicillin–streptomycin (Gibco) in a humid incubator containing 5%CO_2_ at 37°C.

M0 macrophages were divided into three groups: sh-NC (negative control), sh-IL1RN, and sh-IL36G. For lentiviral transfection, 5 × 10^5^ cells/well were seeded and cultured to 70–90% confluence, then infected with lentivirus (MOI = 10, ~5 × 10^6^ TU/mL) and 5 μg/mL polybrene (Merck, USA) for 4 h. After dilution with fresh medium and a further 24 h incubation, cells were replaced with complete medium. Transfection efficiency was verified at 48 h by fluorescence observation, and stable lines were selected using 60 μg/mL puromycin (Gibco, USA). Lentivirus packaging was performed by Sangon Biotech (Shanghai, China). The shRNA sequences used for gene silencing were as follows: sh-IL1RN: 5′-CGAGAACAGAAAGCAGGACAA-3′; sh-IL36G: 5′-CCCATCATTCTGACTTCAGAA-3′; and sh-NC: 5′-CCTAAGGTTAAGTCGCCCTCG-3′.

For co-culture experiments, M0 macrophages from each group were placed in the upper chamber of a Transwell insert (Corning, USA; 0.4 μm pore size), while TE-1 or EC109 cells (1 × 10^5^ cells/well) were seeded in the lower chamber. The system was maintained at 37°C with 5% CO_2_ for 48 h, after which tumor cells were harvested for subsequent analyses.

### Enzyme-linked immunosorbent assay

After 48 h of co-culture between macrophages and EC cells, supernatants were collected and centrifuged at 300 × g for 10 min, then stored at -80°C. IL-10 levels were measured using a human IL-10 ELISA kit (Invitrogen, 88-7106). A standard curve was generated through serial dilutions, and both standards and samples were added to 96-well plates pre-coated with capture antibodies. After sequential incubation with detection antibodies, HRP–streptavidin, and TMB substrate, absorbance was recorded at 450 nm. IL-10 concentrations (pg/mL) were calculated based on the standard curve and reported as mean ± SEM.

### Real-time quantitative polymerase chain reaction

Total RNA was extracted from cells using Trizol (16096020, Thermo Fisher Scientific, New York, USA). mRNA was reverse transcribed with the PrimeScript RT kit (Takara, Japan), and miRNA cDNA was synthesized using the PolyA tail assay kit (Sangon Biotech, China). qPCR reactions were performed with the SYBR Premix Ex Taq II kit (Takara, Japan) on an ABI 7500 Real-Time PCR System (Applied Biosystems, USA). GAPDH acted as an internal reference to the gene. Gene expression levels were quantified using the 2^⁻ΔΔCt^ method. Primer sequences are listed in **Supplementary Table S1**.

### Western blot analysis

Total proteins were extracted, separated by SDS–PAGE, and transferred onto PVDF membranes (1620177, BIO-RAD, USA). Membranes were blocked with 5% skim milk or 5% BSA for 1 h at ambient temperature and incubated overnight at 4 °C with primary antibodies against IL1RN (IL-1RA, ab303490, 1:1000), IL36G (ab239526, 1:1000), E-cadherin (ab231303, 1:1000), N-cadherin (ab76011, 1:1000), and Vimentin (ab8978, 1:1000) (all from Abcam, UK), as well as GAPDH (2118, 1:5000, Cell Signaling Technology, USA). After washing, membranes were incubated with HRP-conjugated goat anti-rabbit IgG (ab6721, 1:5000, Abcam, UK) for 1 h at ambient temperature. Protein bands were visualized using ECL substrate (1705062, Bio-Rad, USA) and imaged with the ImageQuant LAS 4000C system (GE, USA). GAPDH served as the loading control, and protein expression levels were quantified by the ratio of target to GAPDH band intensity.

### CCK-8 assay

Cell proliferation was assessed using the CCK-8 kit (K1018, Apexbio, USA). Cells (1 × 104/well) were seeded into 96-well plates (100 μL/well) and incubated with 10 μL CCK-8 solution for 2 h at 37°C at each time point (days 1, 2, 3, and 4). Absorbance was measured at 450 nm using a microplate reader, and proliferation curves were generated based on absorbance values.

### Cell scratch assay

Cells (5 × 10^5^/well) were seeded into 6-well plates and cultured for 48 h. Linear scratches were created using a 200 μL sterile pipette tip, and the medium was replaced with serum-free medium. Images were captured at 0 h and 48 h using an optical microscope (Leica DM500). Scratch width was quantified using ImageJ software, and cell migration ability was evaluated by comparing the relative reduction in scratch width between groups.

### Transwell assay

Cell migration and invasion were evaluated using Transwell chambers (354480, Shanghai Yanhui Biotechnology Co., Ltd., China). For invasion assays, ECM Matrigel (EHS matrix, E1270-1ML, Sigma, USA) was diluted to 1 mg/mL in serum-free medium (1:9) and coated (40 μL/chamber) onto the upper surface of the polycarbonate membrane, followed by incubation at 37°C, 5% CO_2_ for 5 h to allow gel polymerization. The gel was then rehydrated with 70 μL serum-free DMEM for 30 min. After 24 h of serum starvation, cells were digested, centrifuged, and resuspended in serum-free DMEM at 2.5 × 10^5^ cells/mL. A total of 200 μL cell suspension was added to the upper chamber, while 700 μL DMEM containing 10% FBS was placed in the lower chamber as a chemoattractant. Following 24 h incubation at 37°C, non-invading cells were removed from the upper surface using a cotton swab, and the membranes were fixed with methanol for 30 min and stained with crystal violet for 20 min. Cells that had migrated or invaded to the lower surface were imaged and quantified in five randomly selected fields under an inverted microscope. For migration assays, the same procedure was followed except that the Matrigel coating step was omitted.

### Animal experiment

Thirty-six 6-week-old BALB/c nude mice (20 ± 2 g; Beijing Vital River Experimental Animal Technology Co., Ltd., China) were maintained under standard conditions (23 ± 1°C, 12 h light/dark cycle) with free access to food and water. All animals were acclimated for one week prior to experimentation. All procedures were approved by the Animal Ethics Committee of the Second Affiliated Hospital of Nanchang Medical College.

A total of 1 × 10^6^ EC109 cells (100 μL) were subcutaneously injected into nude mice. Once tumors had formed, mice were randomly divided into three groups (n = 6 per group): sh-NC, sh-IL1RN, and sh-IL36G groups. Each group received multiple intra-tumoral injections of the corresponding lentiviral solution (titer ≈ 5 × 10^7^ TU/mL, 100 μL per injection) once per week for four consecutive weeks. The sh-NC group received control lentivirus. Tumor dimensions were measured weekly using a Vernier caliper, including the short diameter (a) and long diameter (b). Tumor volume was calculated using the formula π(a^2^b)/6. After 28 days, mice were anesthetized with pentobarbital sodium (100 mg/kg, P3761, Sigma). Tumors were excised and weighed using an analytical balance.

For the lung metastasis model, luciferase-labeled EC109 cells (1 × 10^6^) were injected via the caudal vein. Grouping and treatment followed the same protocol as above. Bioluminescence imaging (BLI) was performed using an IVIS Spectrum system (PerkinElmer, USA) after intraperitoneal injection of D-luciferin (150 mg/kg). BLI data were analyzed using Living Image software v2.50, and metastatic burden was quantified based on photon flux within manually defined lung regions of interest.

### Hematoxylin and eosin staining

Tumor tissues from nude mice were fixed in 10% neutral formalin, embedded in paraffin, and sectioned. Sections were deparaffinized in xylene, rehydrated through a graded ethanol series, and stained sequentially with H&E. After dehydration with xylene and fixation with neutral resin, the stained sections were examined under an optical microscope.

### Immunohistochemistry

Paraffin-embedded tumor and lung sections were immunostained with Ki-67 (ab15580, 1:200), E-cadherin (ab231303, 1:250), N-cadherin (ab76011, 1:200), and Vimentin (ab8978, 1:200), all from Abcam (Cambridge, UK). Secondary antibodies, including goat anti-rabbit IgG (ab6721, 1:5000) and goat anti-mouse IgG (ab205719, 1:5000), were also obtained from Abcam. Color development was performed using a DAB kit (P0203, Beyotime, Shanghai, China), and samples were visualized under an upright microscope (BX63, Olympus, Japan).

### Statistics

All statistical analyses were performed using R software (version 4.2.1; https://www.r-project.org/) with RStudio serving as the integrated development environment. Perl (version 5.30.0; https://www.perl.org/) was used for data preprocessing, and Cytoscape (version 3.7.2; https://cytoscape.org/) was used for network visualization. Quantitative data are presented as mean ± standard deviation (SD). Comparisons between two groups were conducted using independent-sample *t-*tests, whereas comparisons among multiple groups were analyzed by one-way analysis of variance (ANOVA) followed by Tukey’s *post hoc* test. For longitudinal measurements, repeated-measures ANOVA with Tukey’s correction was applied. Statistical significance was defined as p < 0.05.

## Results

### scRNA-seq analysis reveals extensive transcriptional heterogeneity in EC tissues

scRNA-seq data from seven EC samples in the GSE145370 dataset were processed with the Seurat package in R. Following quality control and normalization, low-quality cells were excluded using the thresholds nFeature_RNA > 200, nCount_RNA > 1000, nCount_RNA < 20000, and percent.mt < 20 (**Supplementary Figure S1A**). Correlation analysis showed r = −0.03 between nCount_RNA and percent.mt, and r = 0.85 between nCount_RNA and nFeature_RNA, confirming high data quality after filtration (**Supplementary Figure S1B**). Highly variable genes (HVGs), defined as genes whose expression variation substantially exceeds technical noise and captures key differences in cell types and cellular states, were subsequently identified. A total of 21,240 genes were included in the variance analysis, and the top 2,000 HVGs ranked by variance were selected for downstream analyses (**Supplementary Figure S1C**).

### PC analysis of scRNA-seq from EC samples

**Figure 1:**
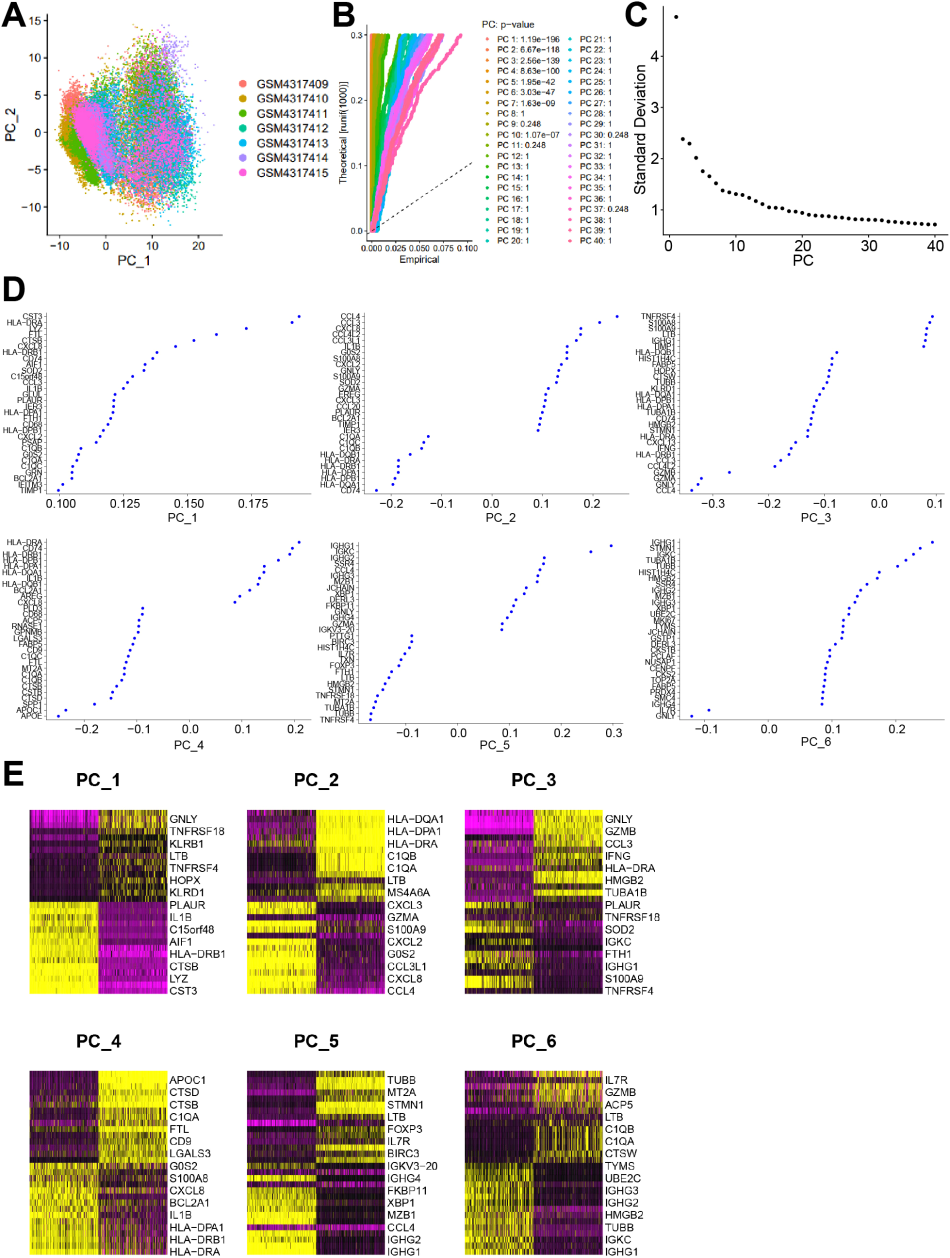
PCA analysis for scRNA-Seq data of esophageal cancer tissue samples. **(A)** Cell cluster in esophageal cancer samples analyzed by PCA. **(B)** The *p* value of the first 40 PCs determined following PCA. **(C)** ElbowPlot analysis identified 20 PCs for tSNE cluster analysis. **(D)** Gene composition in the first six PCs analyzed by PCA. **(E)** A heat map of gene expression in the first six PCs analyzed by PCA.

PCA of the top 2,000 HVGs was conducted using the RunPCA function in the Seurat package, revealing no evident batch effects across the seven EC samples (**Figure 1A**). Significant PCs were identified using the JackStraw resampling approach, in which 1% of the data were randomly permuted and PCA was repeated iteratively to generate a null distribution of feature scores. PCs showing a high proportion of low p-values were considered informative, as illustrated by the JackStrawPlot (**Figure 1B**). Further combined with the ElbowPlot function, the first 20 PCs were selected for subsequent t-SNE analysis (**Figure 1C**). The main contributing genes for the first six PCs were identified (**Figure 1D**), and their expression patterns were visualized using DimHeatmap (**Figure 1E**). Furthermore, the PCA principal components derived from these HVGs showed strong statistical relevance, collectively highlighting the marked transcriptional heterogeneity present within EC tissues.

### Immune cells exhibit distinct malignant states across EC samples

**Figure 2:**
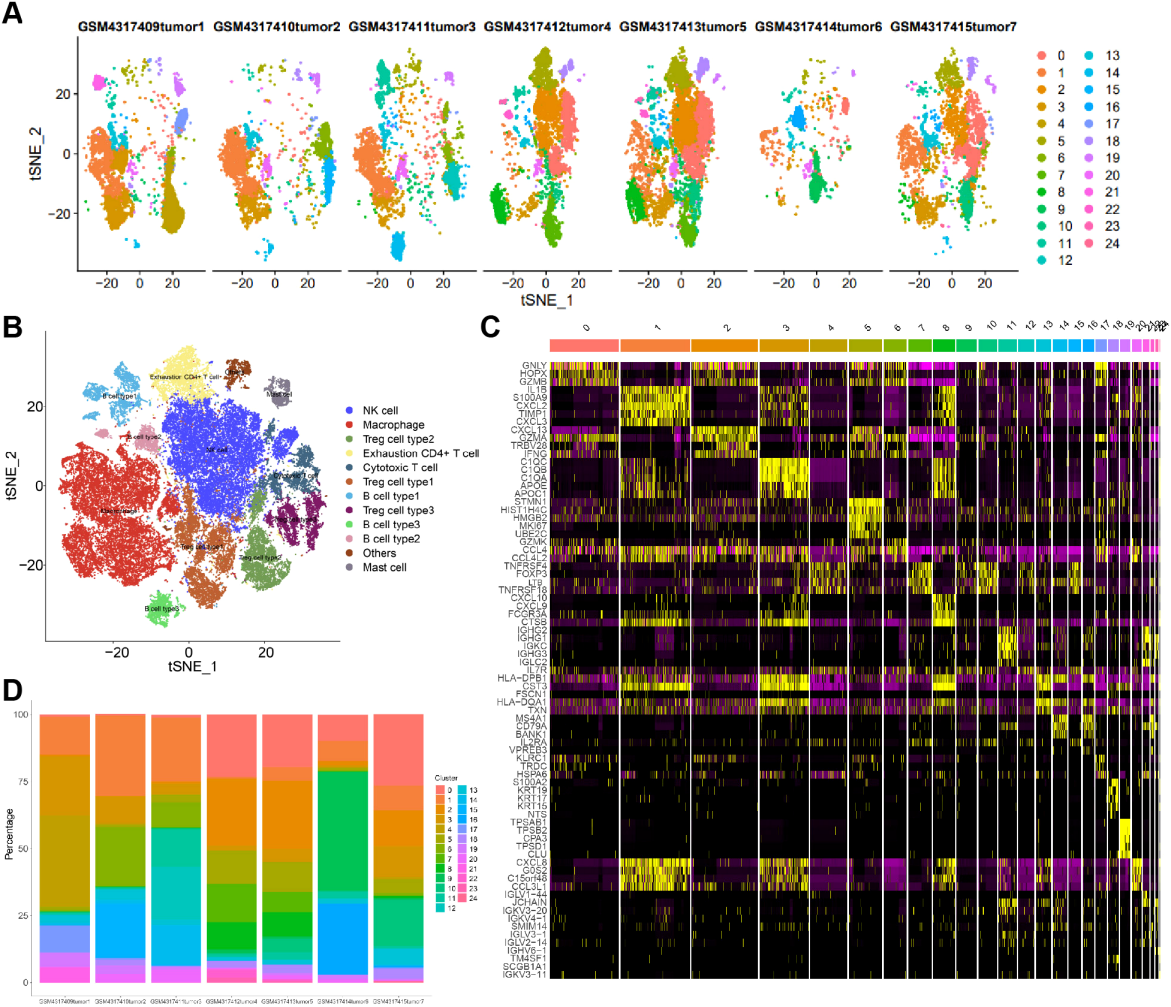
The tSNE clustering analysis of single-cell RNA-seq data from esophageal cancer tissue samples was performed, along with cell annotation. **(A)** Separate display of sample source of each cell cluster of tSNE cluster. **(B)** The 25 cell clusters were annotated as 12 cell types. **(C)** The heat map of the top5 expression of the cell cluster marker gene. **(D)** The proportion of each cell type in 7 cancer samples.

T-SNE was applied following PCA to visualize the high-dimensional single-cell data in two-dimensional space while preserving local cellular relationships. A total of 25 distinct clusters were identified (**Supplementary Figure S2A**). After correction of batch effects, cells originating from different samples showed no marked distributional differences within clusters, aside from varying cluster proportions (**Supplementary Figure S2B**). The cellular compositions across individual EC samples demonstrated both shared and sample-specific cell-type patterns (**Figure 2A**).

Cell-type annotation using the SingleR package identified 12 major cell populations (**Figure 2B**). The expression profiles of the top five marker genes for each annotated cell type were visualized (**Figure 2C**). Tumor cells from the same EC sample appeared in multiple clusters, and immune cells from different samples displayed substantial heterogeneity in malignant potential (**Figure 2D**).

**Figure 3:**
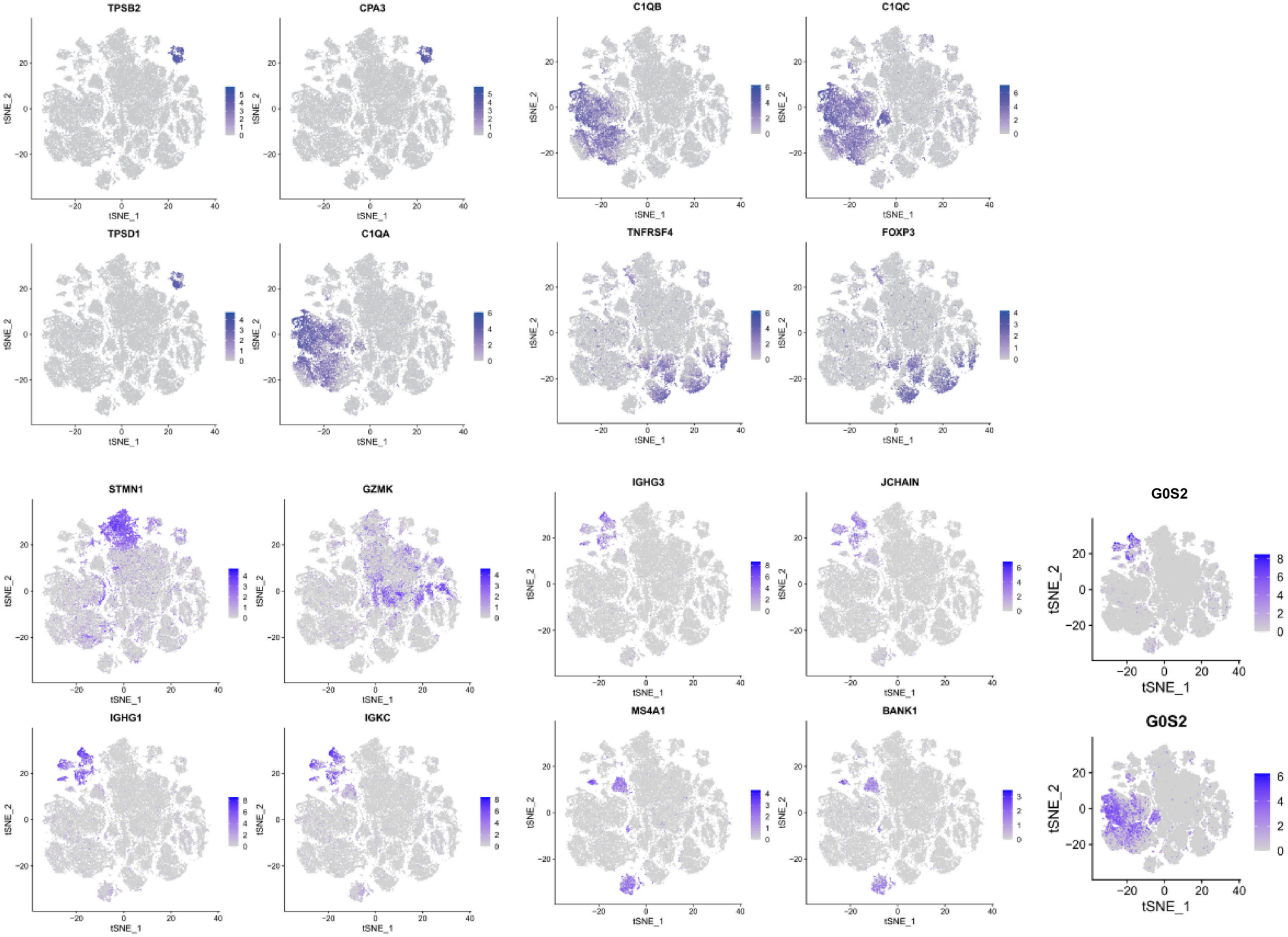
Expression scatter plot **(A)** and violin plot **(B)** of specific marker genes of cell types in all cell clusters.

Cell clusters were further validated using canonical cell-type marker genes. TPSB2, CPA3, and TPSD1 were identified as mast cell markers; IL1B, CXCL2, CXCL8, and GOS2 as macrophage markers; TNFRSF4 and FOXP3 as Treg markers; STMN1 as an exhaustion CD4^+^ T cell marker; GZMK as a cytotoxic T cell marker; IGH2, IGHG1, IGKC, IGHG3, JCHAIN, MS4A1, BANK1, and CD79A as B cell markers, which were further subdivided into B cell type 1 and type 2 based on clustering; and KLRC1 as an natural killer (NK) cell marker (**Figure 3**).

### M2 TAMs dominate the immune microenvironment of EC

**Figure 4:**
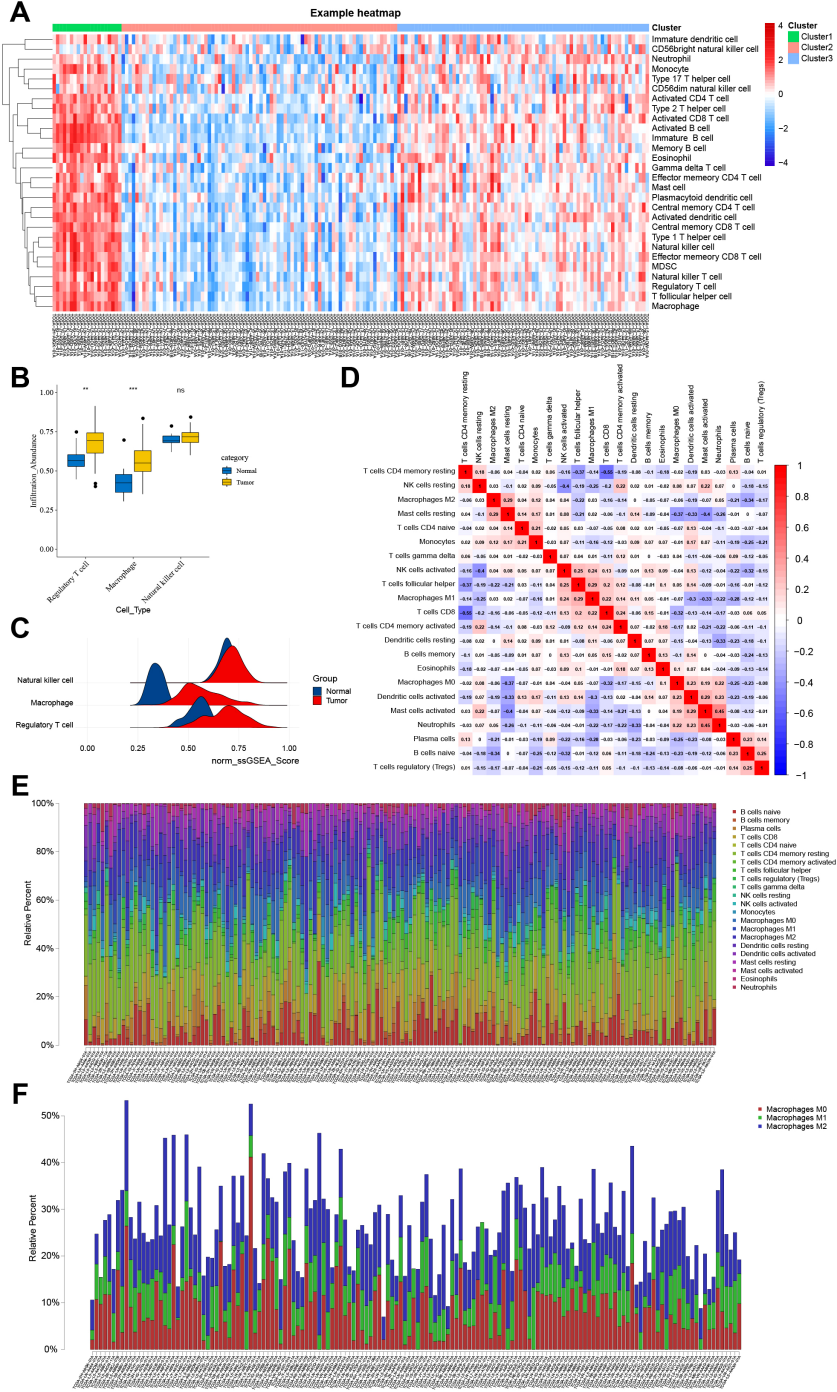
Analysis of immune cell infiltration in the tumor microenvironment based on ssGSEAScore and CIBERSORT algorithms. **(A)** Heat map for cluster analysis of 22 immune cells based on ssGSEAScore. **(B)** Box plot based on the infiltration abundance of ssGSEAScore macrophages, regulatory T cells and natural killer cells. **(C)** Mountain map based on the abundance of ssGSEAScore macrophages, regulatory T cells and natural killer cells. **(D)** Correlation heat map of 22 kinds of immune cell infiltration ratio based on CIBERSORT algorithm. **(E)** Histogram of 22 kinds of immune cell infiltration rate based on CIBERSORT algorithm. **(F)** Histogram of macrophage infiltration rate based on the CIBERSORT algorithm.

Quantitative analysis of scRNA-seq data revealed that macrophages represented the most abundant immune cell population, followed by regulatory T (Treg) cells and NK cells (**Supplementary Table S2**). To further evaluate immune activity, bulk RNA-seq data of EC samples from the TCGA database were analyzed using the GSVA package to perform ssGSEA on 22 immune cell–related functional signatures. Samples were stratified into high-, medium-, and low-immunity clusters, and their enrichment patterns were visualized in a heatmap (**Figure 4A**).

Comparison of ssGSEA scores for macrophages, Treg cells, and NK cells indicated that macrophages exhibited the highest immune infiltration across EC samples (**Figures 4B, C**). The CIBERSORT algorithm was applied to quantify the relative proportions of 22 immune cell subsets in TCGA-ESCA samples, using 1,000 permutations and retaining only results with *p* < 0.05. Correlation heatmaps and bar plots demonstrated that neutrophils, eosinophils, mast cells, dendritic cells, and macrophages were the most enriched immune components within EC tissues (**Figures 4D, E**). Further comparison of macrophage subset infiltration demonstrated that M2 macrophages constituted the predominant TAM phenotype within the TME (**Figure 4F**). Therefore, subsequent analyses focused on elucidating the biological functions of M2 macrophages in EC.

### Correlation between M2 macrophage infiltration and patient prognosis in EC

**Figure 5:**
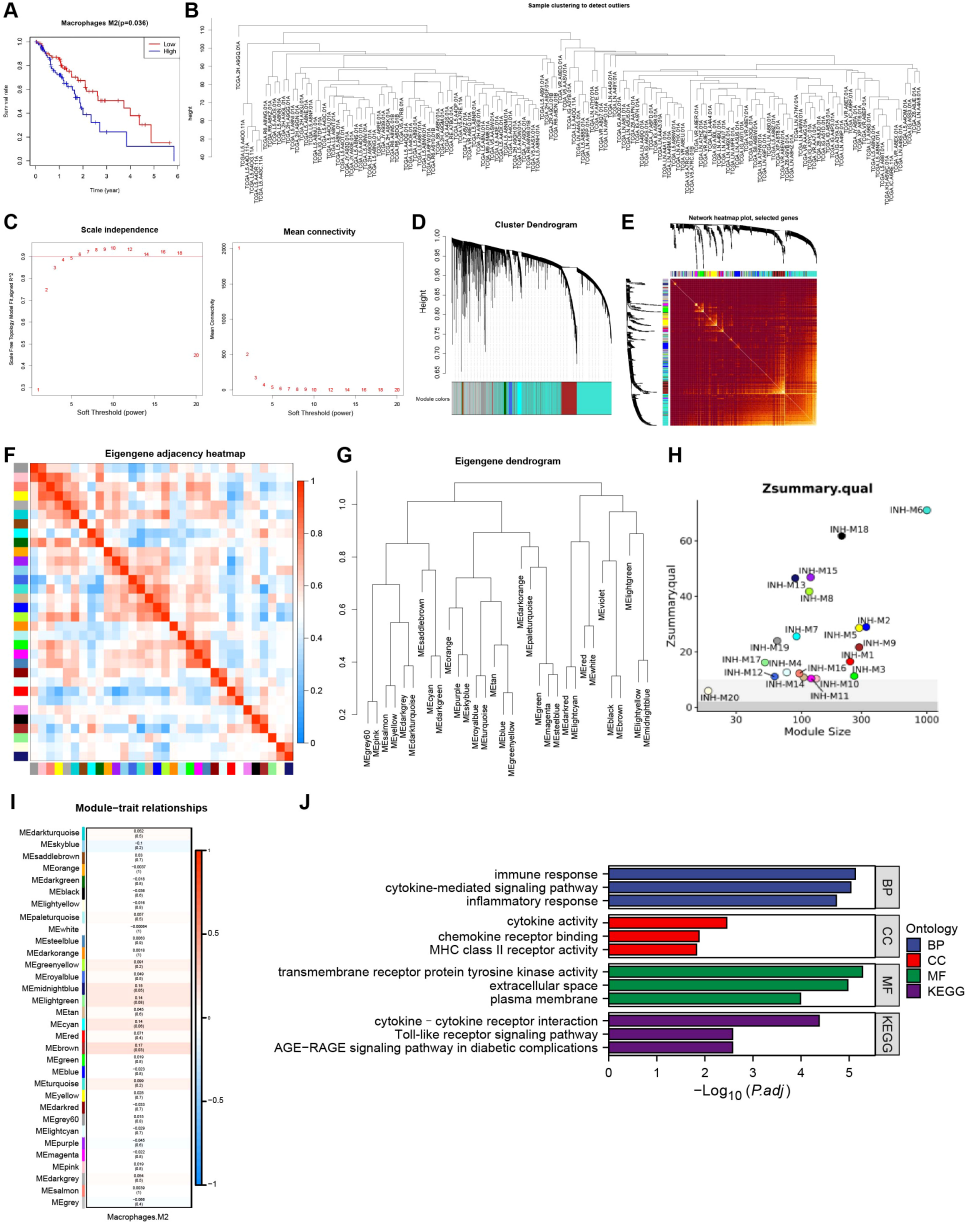
Analysis of M2 macrophage marker genes in esophageal cancer samples using WGCNA. **(A)** Kaplan-Meier analysis of the correlation between the prognosis of patients with esophageal cancer and the content of M2 macrophages(Tumor group, n=162). **(B)** Cluster diagram of TCGA-ESCA dataset sample. **(C)** Soft threshold power analysis was used to obtain the scale-free fit index of the network topology. **(D)** Gene co-expression network was constructed by WGCNA, and each color represents a module in the gene co-expression network constructed by WGCNA. **(E)** Co-expressed gene interaction analysis, the different colors on the horizontal axis and the vertical axis represent different modules, the yellow brightness in the middle indicates the degree of connection of different modules, and there is no significant difference in the interaction between the different modules, indicating that the scale independence among modules was high. **(F)** Correlation analysis of different modules. **(G)** Cluster analysis of different modules. **(H)** Zsummary statistics of module preservation analysis; **(I)** Correlation between different gene co-expression modules and the ssGSEA score of M2-type macrophages. **(J)** Functional enrichment analysis results for GO and KEGG pathways of genes in the brown module are presented in the revised manuscript.

To evaluate the prognostic significance of M2 macrophages in EC, Cox proportional hazards regression was used to evaluate the relationship between M2 macrophage abundance and overall survival (OS). Kaplan–Meier survival analysis revealed that patients with elevated M2 macrophage infiltration had significantly worse outcomes(**Figure 5A**).

To further explore the molecular characteristics of M2 macrophages, a WGCNA was conducted using the TCGA-ESCA transcriptomic dataset comprising 13,461 genes. The top 25% of genes ranked by expression standard deviation were selected for network construction. Sample clustering confirmed that all samples met quality criteria and showed minimal evidence of outliers (**Figure 5B**). A soft-threshold power (β) of 4 was determined to ensure scale-free topology (**Figure 5C**). Network parameters were set as follows: minimum module size = 30, deepSplit = 2, and cut height = 0.25, resulting in the identification of 33 co-expression modules, each assigned a distinct color (**Figure 5D**). Correlation analysis among the identified modules revealed largely independent gene expression patterns (**Figure 5E**). The eigengene values of the 33 modules were calculated, and clustering analysis based on eigengene correlations was performed to determine expression similarity among modules (**Figures 5F, G**). To assess module robustness, random resampling was applied to a subset of the dataset. All modules exhibited Z-scores greater than 2, indicating strong preservation and stability within the network(**Figure 5H**). Among the identified modules, only the brown module demonstrated a significant and strongest positive correlation with the M2 macrophage ssGSEA score (*P* < 0.05; **Figure 5I**). GO and KEGG pathway enrichment analyses revealed that genes within the brown module were predominantly associated with inflammatory responses and cytokine-mediated signaling pathways, supporting their functional relevance to M2 macrophage infiltration (**Figure 5J**). Therefore, genes from the brown module were selected for subsequent analyses.

### KEGG and GO function enrichment analysis of M2 macrophage marker genes

**Figure 6:**
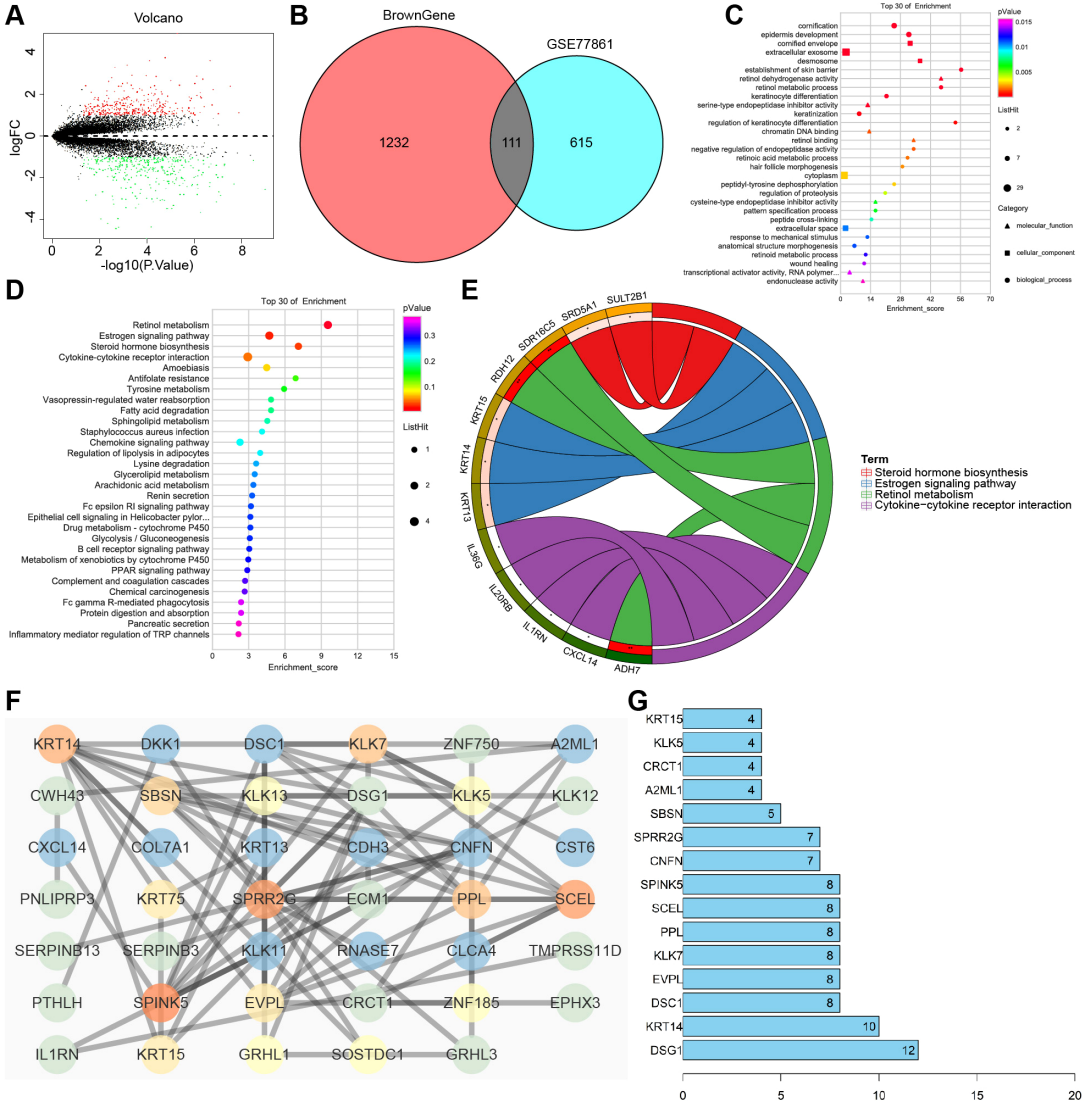
Function enrichment analysis and PPI network construction for M2 macrophage marker genes. **(A)** Volcano map of the DEGs in the microarray GSE77861 (Normal, N = 7, Tumor, N = 7). **(B)** Venn map of the intersection of the Brown module gene and the DEGs in the microarray GSE77861. **(C)** Bubble chart of GO enrichment analysis of 111 intersection genes. **(D)** Bubble chart of KEGG enrichment analysis of 111 intersection genes. **(E)** Circle plot of KEGG enrichment analysis of 111 intersection genes. **(F)** The protein interaction network diagram constructed by Cytoscape software, the color from red to blue indicates that the Degree value and the Combined score value are from small to large. **(G)** The first 15 genes of the Degree value.

WGCNA analysis showed that the Brown module gene had the highest correlation with M2 macrophage infiltration in EC, which contained 1304 genes. To further identify functionally relevant genes, DEGs from the GEO dataset (GSE77861) were intersected with these M2 macrophage–associated genes, yielding 111 overlapping candidates (**Figures 6A, B**). GO and KEGG analyses revealed that these overlapping genes were mainly enriched in BP, such as cornification, epidermal development, and signal transduction, as well as pathways including cytokine–cytokine receptor interaction, retinol metabolism, estrogen signaling, and steroid hormone biosynthesis (**Figures 6C–E**).

A PPI network was constructed using STRING and subsequently visualized in Cytoscape (**Figure 6F**). Based on node degree ranking, the top 15 hub genes were identified: *DSG1, KRT14, DSC1, EVPL, KLK7, PPL, SCEL, SPINK5, CNFN, SPRR2G, SBSN, A2ML1, CRCT1, KLK5, and KRT15* (**Figure 6G**). By integrating these hub genes with key pathway-enriched genes (*RDH12, ADH7, SDR16C5, KRT13, KRT14, KRT15, SRD5A1, SULT2B1, CXCL14, IL20RB, IL1RN*, and *IL36G*), a total of 25 candidate genes were obtained for subsequent analysis (**Figure 6E**).

### Four M2 macrophage–associated genes serve as prognostic biomarkers in EC

**Figure 7:**
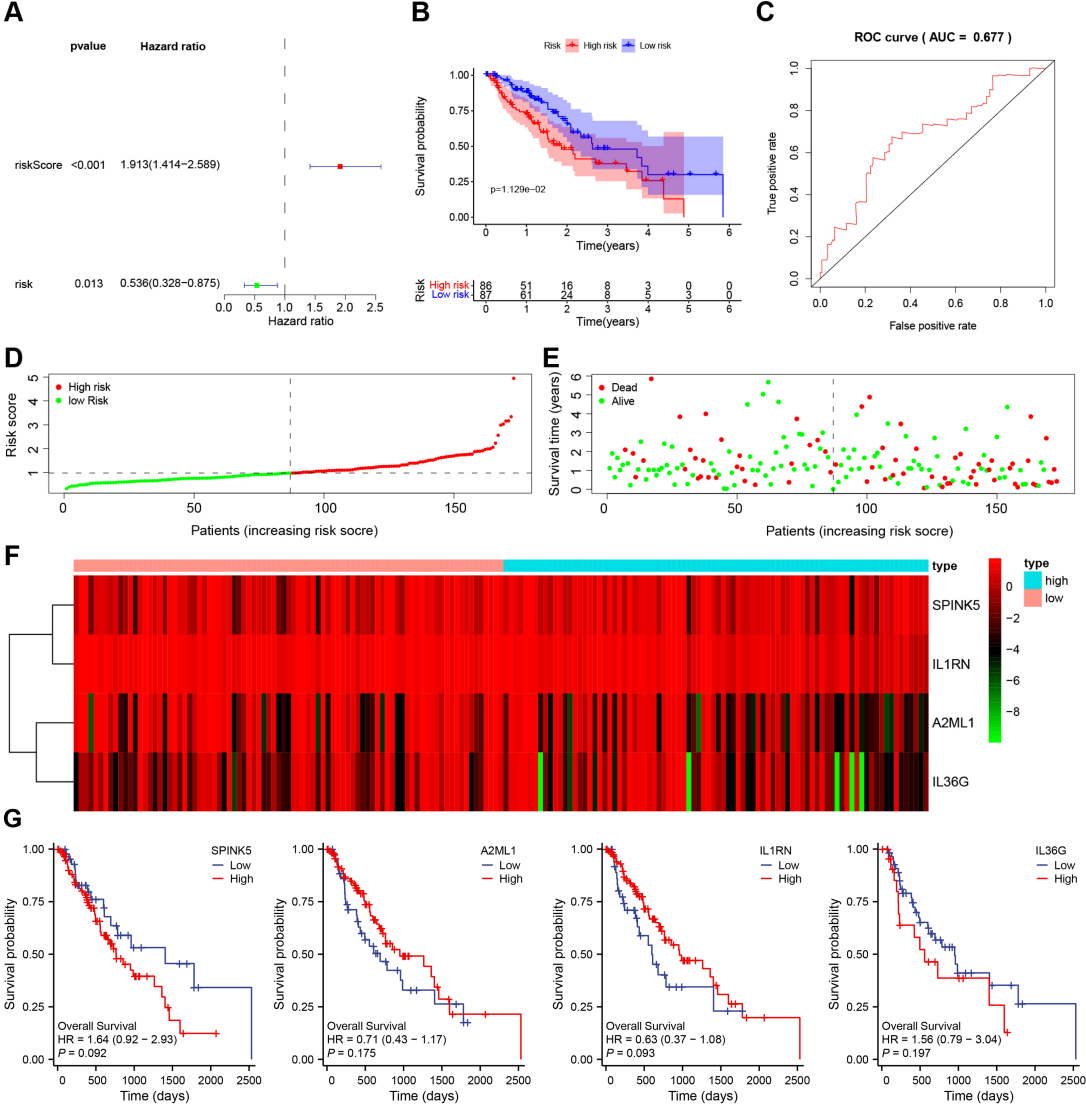
Screening of M2 macrophage marker genes related to the prognosis of patients with esophageal cancer. **(A)** Multivariate Cox regression analysis used to construct the prognostic model. riskScore represents the continuous score based on the expression of the four genes (*SPINK5*, *A2ML1*, *IL1RN*, *IL36G*), and its hazard ratio (HR) reflects the effect of a one-unit increase in score on patient mortality risk. risk represents the categorical grouping variable (high vs. low risk) derived from the riskScore, and its HR compares survival differences between the two groups. **(B)** Survival curve of a risk prognostic model constructed based on 4 M2 macrophage marker genes. **(C)** ROC curve of a risk prognosis model constructed based on 4 M2 macrophage marker genes. **(D)** Risk curve diagram. **(E)**, Survival status diagram. **(F)** Heat map of expression of 4 M2 macrophage marker genes in high and low risk groups. The values in the graph have been normalized. **(G)** Correlation between the four genes and overall survival of esophageal cancer patients.

To identify M2 macrophage–associated genes with prognostic relevance in EC, a multivariate Cox regression model was constructed and optimized using bidirectional stepwise regression. Finally, 4 genes (*SPINK5, A2ML1, IL1RN, and IL36G*) were obtained (**Figure 7A**). A four-gene prognostic risk model was subsequently developed. The risk score (riskScore) was calculated as a continuous variable based on gene expression levels, and patients were stratified into high- and low-risk groups according to the median riskScore. Kaplan–Meier analysis demonstrated that patients in the low-risk group exhibited significantly longer OS than those in the high-risk group (**Figure 7B**). The riskScore was computed using the following formula: Risk Score = (0.67) * *SPINK5* + (-0.54) * A2ML1 + (-0.84) * A2ML1 +IL1RN (0.41) * IL36G.

ROC analysis revealed that the area under the ROC curve (AUC) of the risk model was 0.677 (95% CI: 0.612–0.742) (**Figure 7C**). Multivariate Cox analysis confirmed that the four-gene signature remained an independent prognostic factor after adjustment for clinical covariates (**Supplementary Table S3**). The distribution of risk scores and corresponding survival status is shown in **Figure 7D** and **Figure 7E**, respectively, and **Figure 7F** illustrates the differential expression patterns of the four genes between risk groups. The individual survival contributions of each gene are presented in **Figure 7G**.

Collectively, these results demonstrate that the four-gene M2 macrophage–related signature (*SPINK5, A2ML1, IL1RN*, and *IL36G*) serves as a robust predictor of EC prognosis. Interestingly, although *SPINK5* and *A2ML1* are traditionally characterized as epithelial-associated genes, their strong correlation with M2 macrophage infiltration suggests a potential role in immune–epithelial crosstalk, further underscoring their relevance in the TME.

### Silencing IL1RN and IL36G in macrophages promoted M2 polarization and suppressed EC cell malignancy

**Figure 8:**
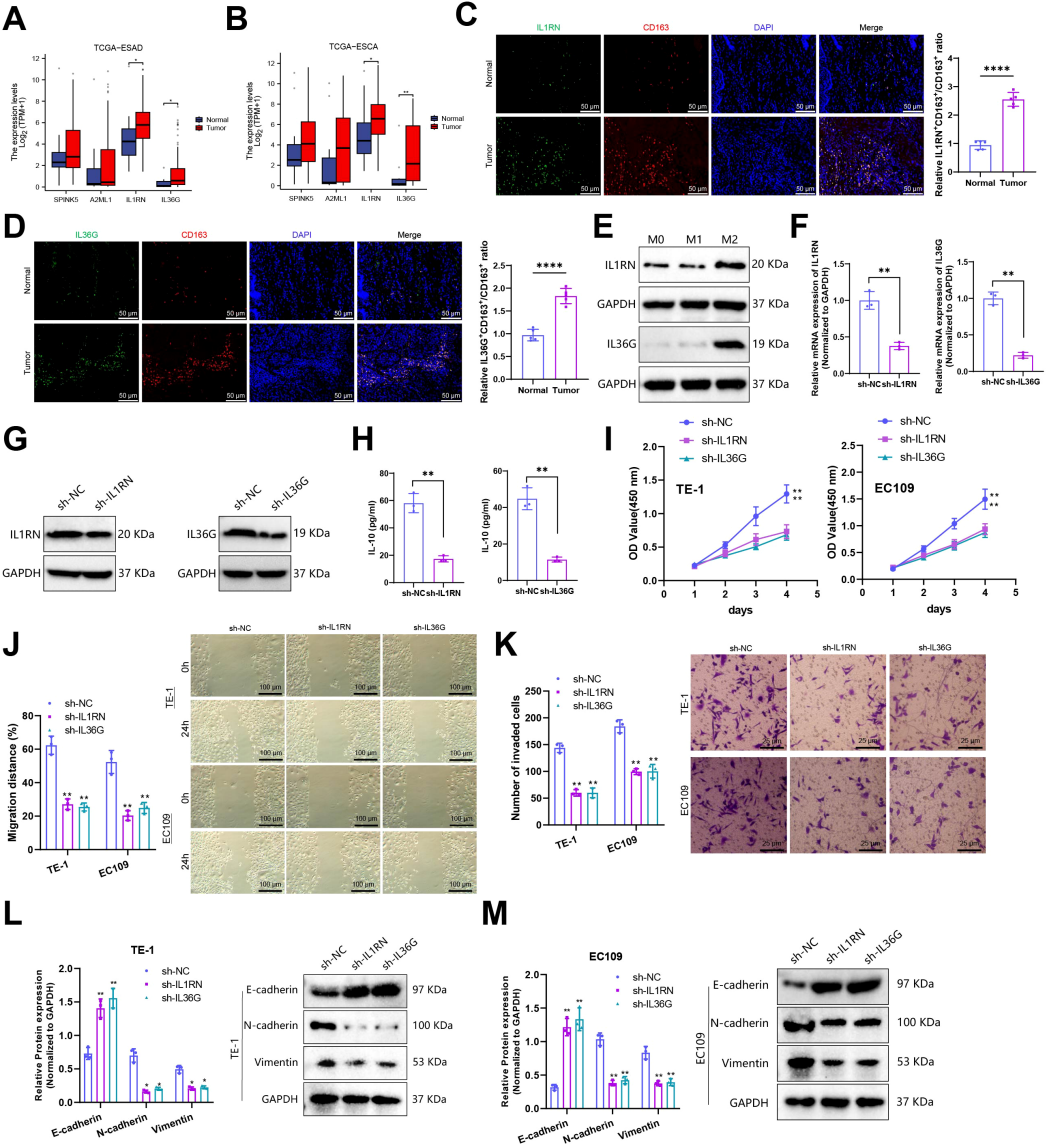
Silencing of *IL1RN* and *IL36G* in macrophages inhibited the malignant phenotype of co-cultured esophageal cancer cells. **(A, B)** Expression levels of SPINK5, A2ML1, IL1RN, and IL36G in TCGA-ESCA and TCGA-ESAD (*P < 0.05, *P < 0.01). **(C, D)** Immunofluorescence staining of clinical samples showing IL1RN **(C)** and IL36G **(D)** expression and colocalization with M2 macrophage markers (Normal = 5; Tumor = 5). **(E)** Western blot analysis of IL1RN and IL36G expression in macrophage subtypes. F–G, RT-qPCR **(F)** and Western blot **(G)** validation of shRNA knockdown efficiency for IL1RN and IL36G in macrophages. **(H)** ELISA analysis of IL-10 secretion levels in macrophages (****P < 0.0001). **(I)** Following co-culture, CCK-8 was used to detect the proliferation of TE-1 and EC109 cells after silenced IL1RN and IL36G. **(J)** Migration and invasion capacity of TE-1 and EC109 cells after silencing IL1RN and IL36G by cell scratch assay after co-culture. **(K)** Transwell assay was used to detect the invasion ability of TE-1 and EC109 cells after silencing IL1RN and IL36G following co-culture. **(L, M)** Expression of N-cadherin, Vimentin, and E-cadherin in TE-1 cells and EC109 cells silenced IL1RN and IL36G were detected by Western blot after co-culture. *P < 0.05, compared with sh-NC group. **P < 0.01, compared with sh-NC group. All cell experiments were independently repeated three times. The symbol “*” indicates P < 0.05, representing statistical significance between groups.

To validate the roles of M2-like TAM–related genes in EC progression, we examined the expression of the four genes (*SPINK5*, *A2ML1*, *IL1RN*, and *IL36G*) in tumor and normal tissues based on TCGA-ESCA data. Among them, IL1RN and IL36G were significantly upregulated in tumor tissues and selected for further investigation (**Figure 8A**). Similar expression patterns were observed in TCGA-ESAD, where IL1RN and IL36G were also elevated in EAC, whereas SPINK5 and A2ML1 displayed no significant differences between tumor and normal tissues (**Figure 8B**). Immunofluorescence staining of clinical EC samples further demonstrated co-expression of *IL1RN* and *IL36G* with M2 macrophage markers, with both genes showing significantly higher expression in tumor tissues (**Figures 8C, D**).

Human THP-1 monocytes were differentiated into M0 macrophages by treatment with PMA for 24 h. Expression profiling revealed that *IL1RN* and *IL36G* were markedly upregulated in M2 macrophages, while their expression was low in M0 and M1 subtypes (**Figure 8E**).

To investigate their functional roles, two shRNA sequences targeting IL1RN and IL36G were designed and validated for efficient gene silencing in macrophages (**Figures 8F, G**). ELISA analysis showed that IL-10 secretion was reduced in the sh-IL1RN and sh-IL36G groups relative to the sh-NC control (**Figure 8H**). Subsequently, macrophages from each group were co-cultured with TE-1 and EC109 cells, and CCK-8 (**Figure 8I**), wound-healing (**Figure 8J**), and Transwell assays (**Figure 8K**) were performed to evaluate changes in the proliferative, migratory, invasive, and metastatic capacities of TE-1 and EC109 cells. The results showed that co-culture with macrophages in which IL1RN and IL36G were silenced significantly attenuated the proliferation, migration, invasion, and metastatic potential of both TE-1 and EC109 cells. Furthermore, the expression of epithelial-mesenchymal transition (EMT)-related genes in esophageal cancer cells was examined. Compared with the sh-NC group, TE-1 and EC109 cells co-cultured with macrophages lacking IL1RN and IL36G exhibited significantly reduced expression of N-cadherin and Vimentin, accompanied by a marked increase in E-cadherin expression (**Figures 8L, M**). Together, these findings suggest that silencing *IL1RN* and *IL36G* inhibits macrophage M2 polarization, thereby reducing EC cell proliferation, invasion, migration, and EMT progression.

### Silencing *IL1RN* and *IL36G* suppressed tumor growth and metastasis in EC

**Figure 9:**
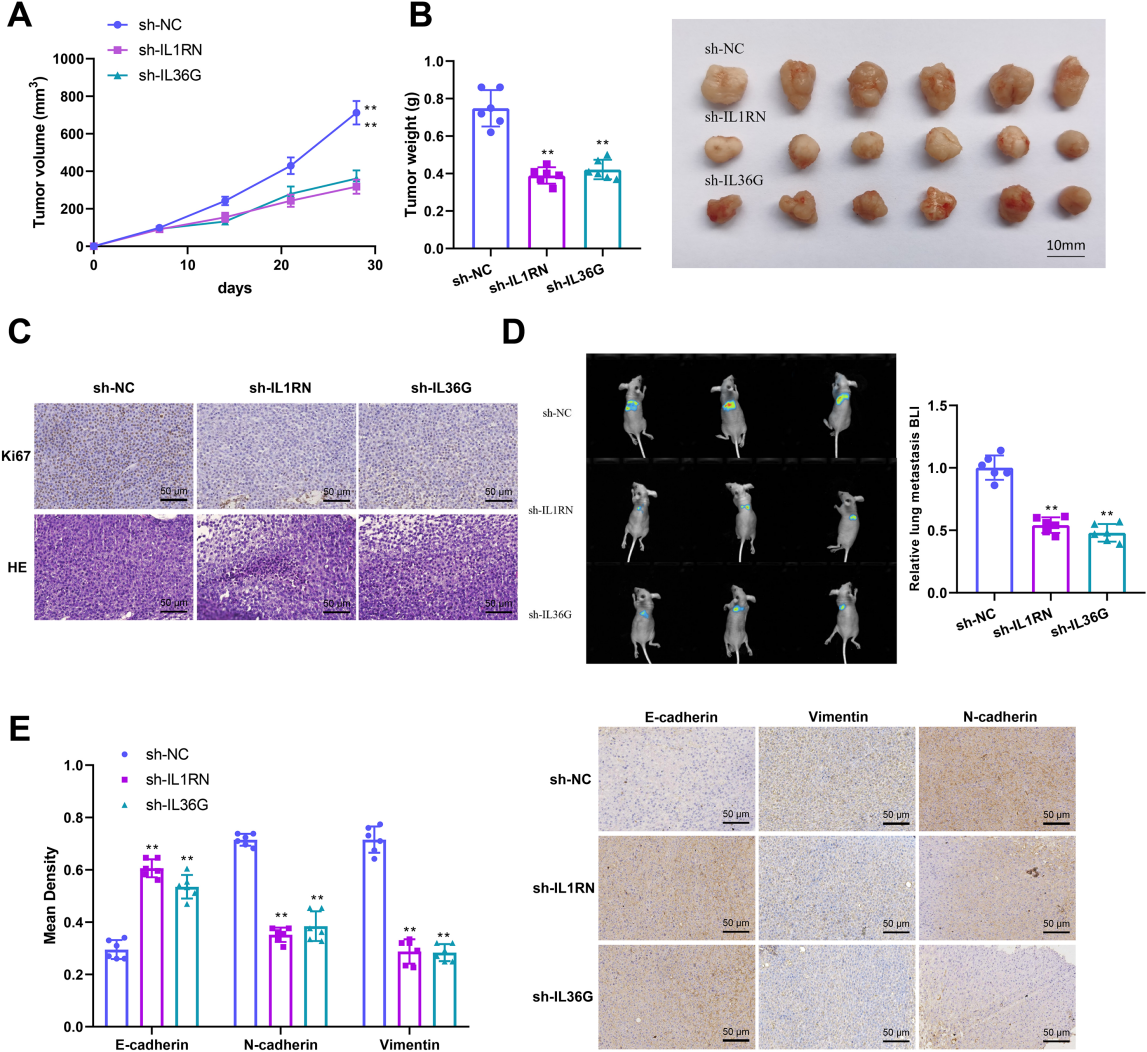
Silencing *IL1RN* and *IL36G* inhibited tumor growth and metastasis in esophageal cancer. **(A)** Tumor growth curve of nude mice. **(B)** Representational images and weight statistics of nude mice. **(C)** ki-67 and H&E staining of tumor tissue in nude mice. **(D)** IVIS image of lung tissue and BLI result of lung tissue in nude mice. **(E)** The expression of E-cadherin, Vimentin and N-cadherin in lung tissues of nude mice was observed by IHC staining. **P < 0.01, compared with sh-NC group. N = 6/per group.

Given the *in vitro* evidence that IL1RN and IL36G silencing in macrophages attenuates the malignant behavior of EC cells, we next evaluated their effects *in vivo* using xenograft mouse models. Nude mice were subcutaneously injected with EC109 cells transduced with sh-NC, sh-IL1RN, or sh-IL36G lentiviruses. Tumor growth was monitored throughout the experiment, and both tumor volume and tumor weight were significantly reduced in the sh-IL1RN and sh-IL36G groups compared with the control group (**Figures 9A, B**).

In addition, IHC staining showed that silencing IL1RN and IL36G decreased the expression of proliferative marker ki-67 (**Figure 9C**). H&E staining showed that control tumors exhibited densely packed cells with typical morphology, whereas tumors in the sh-IL1RN and sh-IL36G groups displayed reduced cellular density, nuclear condensation, and increased necrosis (**Figure 9C**). Subsequently, we established a tumor metastasis model by injecting EC cells into the tail vein of nude mice. Bioluminescence images showed that the intensity of bioluminescence in the lung tissue of nude mice was reduced after silencing IL1RN and IL36G, indicating fewer metastatic tumor cells (**Figure 9D**). Further, IHC staining of lung tissues revealed that IL1RN and IL36G silencing downregulated Vimentin and N-cadherin expression while upregulating E-cadherin, indicating suppression of EMT (**Figure 9E**).

Overall, the findings indicate that silencing IL1RN and IL36G suppresses tumor growth and metastatic progression in EC. It should be noted that, because lentiviral manipulation *in vivo* is not cell type-specific, these results primarily reflect the functional roles of *IL1RN* and *IL36G* in overall tumor progression and cannot, by themselves, be taken as definitive evidence of macrophage-specific mechanisms in tumor-associated macrophages.

## Discussion

Combined application of scRNA-seq and bulk RNA-seq enables precise characterization of tumor heterogeneity and elucidation of the immune landscape associated with clinical outcomes (27). Through comprehensive scRNA-seq analysis, we identified four genes (*SPINK5*, *A2ML1*, *IL1RN*, and *IL36G*) that were predominantly expressed in M2 macrophages, suggesting their potential roles in shaping the immunosuppressive tumor microenvironment. WGCNA further demonstrated strong correlations between these genes and M2 macrophage infiltration, underscoring their relevance to immune–tumor cell interactions in EC. A four-gene prognostic signature incorporating *SPINK5, A2ML1, IL1RN* and *IL36G* was constructed and showed substantial predictive value for OS. Functional experiments provided additional support by demonstrating that silencing IL1RN and IL36G reduced EC cell proliferation, migration, and invasion *in vitro* and suppressed tumor growth and metastasis *in vivo*. These results suggest that IL1RN and IL36G play crucial roles in EC progression and may represent viable prognostic and therapeutic targets.

IL1RN and IL36G have been reported as potential therapeutic targets in multiple cancer types. IL1RN encodes interleukin-1 receptor antagonist (IL-1RA), which functions as a tumor suppressor by inhibiting IL-1 signaling. IL-1RA reduces tumor cell proliferation, migration, angiogenesis, and lymphangiogenesis, thereby limiting tumor growth and metastasis. In ESCC, reduced IL-1RA expression is linked to lymph node metastasis and advanced pathological stages. Exogenous IL-1RA (e.g., anakinra) significantly suppresses tumor growth and lymphangiogenesis, supporting the therapeutic potential of IL1RN (28, 29). IL36G, a member of the IL-36 cytokine family, has recently been recognized as an important regulator of the tumor immune microenvironment (TIME) across multiple cancer types. Earlier studies identified IL-36γ primarily as an antitumor cytokine that activates CD8^+^ T, NK, and γδ T cells, thereby enhancing antitumor immunity. However, recent evidence suggests that IL-36 signaling may also exert pro-tumor effects in certain cancers, promoting EMT, cytokine imbalance, and tumor cell survival and migration (30, 31). In this study, IL1RN and IL36G were highly expressed in M2 macrophages, and their silencing in macrophages markedly suppressed the malignant phenotype of tumor cells and inhibited tumor growth *in vivo*, suggesting that these genes may exert pro-tumorigenic functions in EC and represent potential therapeutic targets.

Initial findings from the present study revealed marked tumor heterogeneity and substantial diversity among infiltrating immune cells in EC, with M2 macrophages emerging as key contributors to disease progression. EC is recognized as a highly heterogeneous malignancy that is frequently diagnosed at advanced stages (32). Recent studies have further shown that the composition of tumor-infiltrating immune cells is closely associated with EC progression, prognosis, and therapeutic response (33). Moreover, M2-like TAMs have been associated with unfavorable prognosis of cancers, including EC and triple-negative breast cancer (27, 34). The present investigation similarly demonstrated a significant correlation between M2 macrophage infiltration and survival in EC. Published evidence also indicates that tumor-infiltrating M2 macrophages serve as important predictors of chemotherapy response and survival in patients with EC, further highlighting their clinical relevance (34).

Further analysis in this study revealed that three M2-type macrophage-related genes (*SPINK5, A2ML1, IL1RN)*, and *IL36G* could predict the prognosis of EC patients effectively. Based on TCGA-ESCA data, IL1RN and IL36G were significantly upregulated in both EAC and ESCC tissues compared with normal tissues and were selected for *in vitro* and *in vivo* validation. Experimental results demonstrated that silencing *IL1RN* and *IL36G* in macrophages significantly suppressed the malignant behavior of EC cells and reduced tumor growth and metastasis in animal models. SPINK5, a well-characterized serine protease inhibitor involved in epithelial barrier regulation (35), has been reported to inhibit the malignant phenotype of EC cells by suppressing the Wnt/β-catenin signaling pathway, supporting its potential as a therapeutic target (11). A2ML1, a monomeric protease inhibitor containing histidine residues that mediate hydroxyl transfer reactions (36), has rarely been studied in the context of EC. Although SPINK5 and A2ML1 are not classical immune markers, their strong association with M2 macrophage infiltration suggests that they may function as transcriptional readouts of M2 TAM activity within the TME. Genetic studies have shown that IL1RN polymorphisms influence cancer susceptibility, with variants rs3181052, rs452204, and rs315919 associated with reduced EC risk in the Northwest Han population (37). Accumulating evidence has revealed that polymorphisms in IL36G gene are related to plaque psoriasis (38). Recent data also indicate that IL-36G enhances colony formation, migration, and invasion in gastric cancer cell lines (39).

Although a prognostic model based on M2 macrophage–related genes was successfully established and demonstrated the ability to characterize immune cell heterogeneity and predict clinical outcomes in EC, several limitations remain. The dataset used for model construction was limited, and the multivariate Cox regression model built on four genes (*SPINK5, A2ML1, IL1RN*, and *IL36G*) achieved an AUC of 0.677, indicating only moderate predictive performance. Although the model showed potential in distinguishing between high- and low-risk patients and partially predicting survival outcomes in EC, the AUC value did not reach the ideal standard for clinical application. As a result, the model may be more appropriate as an auxiliary prognostic tool when combined with clinical indicators and pathological features to support individualized treatment planning. In addition, the model was derived from a relatively small cohort, raising concerns regarding potential overfitting and limiting its applicability across diverse patient populations. Therefore, Large-scale, multi-center, and heterogeneous independent cohorts are needed for external validation to assess the reproducibility, robustness, and clinical utility of the model.

Second, the variables included in the current model remain limited, indicating considerable room for further improvement compared with existing prognostic models. For example, Riccardo et al. developed an EC prognostic model with an AUC of 0.63, a level similar to the present study (40). However, models based on large-scale clinical data and machine learning methods, such as the random survival forest (RSF), have achieved 5-year survival prediction AUCs of up to 83.9%, substantially outperforming traditional Cox regression models (41). Comprehensive prognostic systems that integrate clinical, imaging, and molecular features commonly achieve AUC or C-index values ranging from 0.7 to 0.8. For example, a postoperative prognostic model for esophageal squamous cell carcinoma reached a C-index of 0.773, surpassing the accuracy of TNM staging (42). Deep learning frameworks, including ResNet-CBAM, can attain AUC values as high as 0.835 for long-term survival prediction (43). Therefore, the current model could be further optimized in terms of variable selection and structural enhancement.

Third, the current cellular and animal experiments were primarily based on EC cell lines (TE-1 and EC109) and nude mouse xenograft models. Although IL1RN and IL36G silencing reduced tumor growth and metastasis in these systems, validation in primary tumor tissues remains absent. Compared with cell lines and xenograft models, primary tumors contain more complex immune microenvironments and cellular interactions that more accurately reflect the biological characteristics of EC. While *in vitro* and *in vivo* data provide a strong foundation, these models remain limited in their ability to capture tumor heterogeneity, host immune responses, and tumor–host interactions. Therefore, future studies should include immunohistochemistry, *in situ* hybridization, multi-omics analyses, and even CRISPR-based genetic perturbations in primary clinical esophageal tumor samples to further validate the expression patterns and functional roles of IL1RN and IL36G. In addition, although our *in vitro* co-culture systems clearly demonstrated that silencing IL1RN and IL36G in macrophages suppresses the malignant phenotypes of esophageal cancer cells, the *in vivo* xenograft models used in this study do not allow for tumor-associated macrophage-specific genetic manipulation. Therefore, the *in vivo* findings should be regarded as supportive evidence for the functional importance of IL1RN and IL36G in tumor progression, rather than as direct proof of their TAM-specific mechanisms. Future studies employing cell type-specific knockout models or targeted delivery strategies will be necessary to further validate these mechanisms.

To enhance the practical utility and scientific value of the model, several directions should be prioritized in future research. First, increasing the sample size and conducting external validation across multi-center cohorts with greater heterogeneity will be essential for assessing the generalizability and robustness of the four-gene signature, thereby improving its potential for clinical application. Second, integrating additional clinical variables, molecular biomarkers, and radiomic features through advanced machine learning and deep learning approaches (e.g., random forest, ResNet) may strengthen model architecture and predictive performance while providing improved decision support (44). Third, incorporation of epigenetic information, including DNA methylation and histone modifications, may further capture cellular phenotypic and functional heterogeneity and enhance prognostic accuracy. Fourth, the potential applicability of this model to other cancer types should be explored by evaluating whether *SPINK5, A2ML1, IL1RN*, and *IL36G* also function as prognostic markers beyond EC. Although this direction involves substantial technical challenges, it offers the possibility of expanding the model’s versatility and cross-cancer relevance. Fifth, in-depth validation in primary clinical esophageal tumor samples using *in situ* assays and spatial transcriptomic techniques is critical to elucidate the spatial expression and functional status of *IL1RN* and *IL36G*, clarify their roles in the TIME, and establish a foundation for M2 macrophage-targeted precision therapies.

## Conclusion

**Figure 10:**
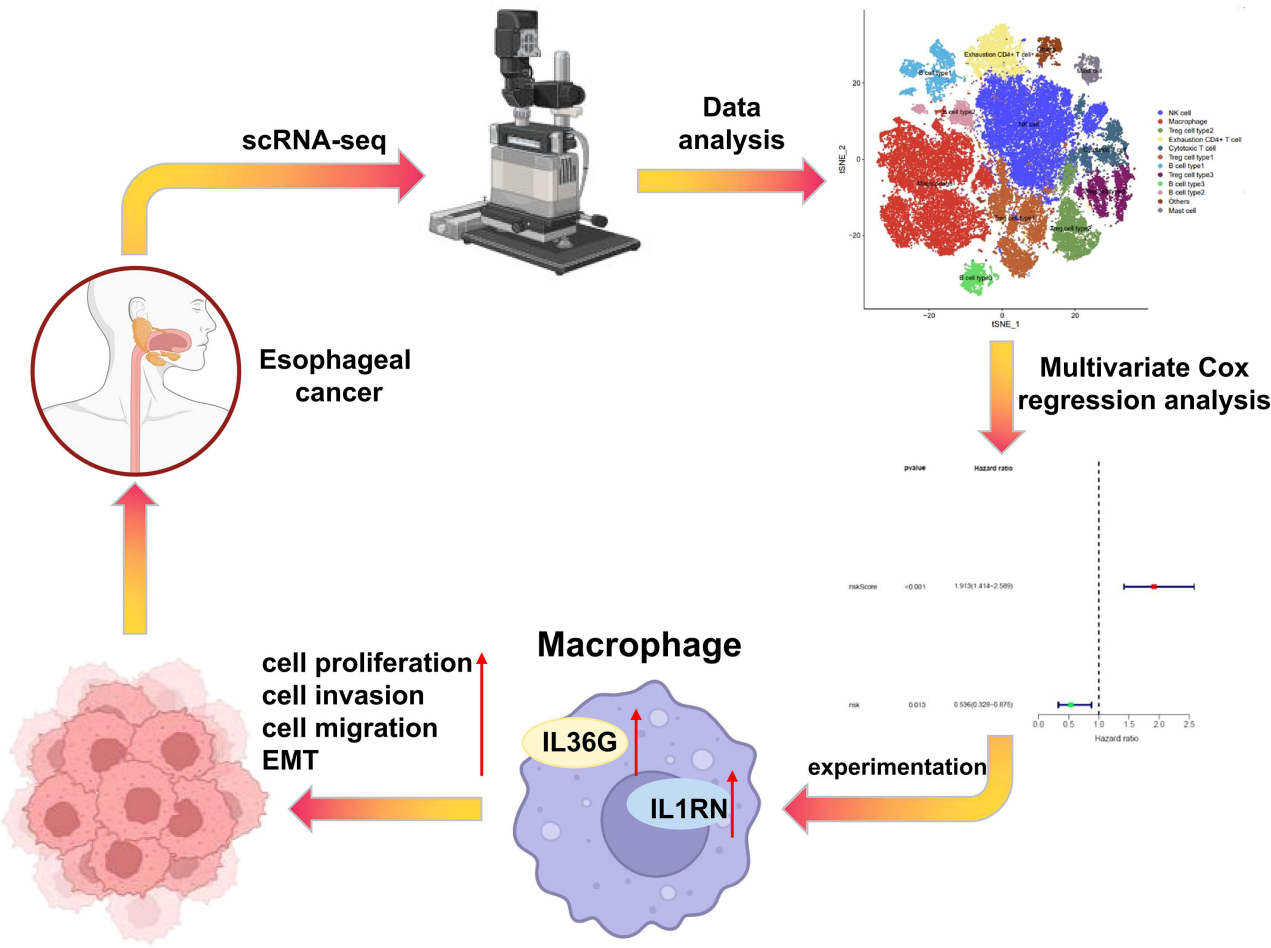
Single-cell RNA-seq combined with Bulk RNA-seq was used to analyze the cell heterogeneity of esophageal cancer and screen M2-type macrophage marker genes to predict the prognosis of patients with esophageal cancer.

Overall, M2-type macrophage-related genes *SPINK5, A2ML1, IL1RN* and *IL36G* appear to be important biological markers for predicting the prognosis of patients with EC, as indicated by their association with suppressed malignant behaviors of EC cells. The gene-based signature derived from these four markers demonstrated strong prognostic accuracy. Functional experiments further showed that silencing *IL1RN* and *IL36G* in macrophages markedly suppressed the malignant behaviors of EC cells and inhibited tumor growth and metastasis *in vivo* (**Figure 10**). These findings provide a mechanistic basis for targeting *IL1RN* and *IL36G* in the development of macrophage-centered therapeutic strategies for EC.

## Data Availability

The authors acknowledge that the data presented in this study must be deposited and made publicly available in an acceptable repository, prior to publication. Frontiers cannot accept a article that does not adhere to our open data policies.

## Supplementary material

Supplementary Figure 1 scRNA-seq analysis of DEGs in esophageal cancer tissue samples. **(A)**, Quality control for the 69756 cells in cancer tissue samples from seven esophageal cancer patients. Three scatter plots respectively represent the number of nFeature_RNA, nCount_RNA and percent.mt in each cell. **(B)**, The correlation between nCount and percent.mt (left), and the correlation between nCount and nFeature (right). **(C)**, Highly variable genes among 21240 genes screened by analysis of variance. Red dots represent highly variable genes and black dots represent invariable genes.

Supplementary Figure 2 tSNE clustering analysis was conducted on the single-cell RNA-seq data of esophageal cancer tissue samples. **(A)**, tSNE cluster analysis clusters cells into 25 cell clusters. **(B)**, Sample source of each cell cluster of tSNE cluster.
