# Supplementary figures and images for: M2 macrophage infiltration drives tumor progression and identifies a multigene prognostic signature in esophageal cancer
Source: Front Immunol. 2026 Feb 2;16:1659048. doi: 10.3389/fimmu.2025.1659048 (PMC12907416; doi:10.3389/fimmu.2025.1659048)

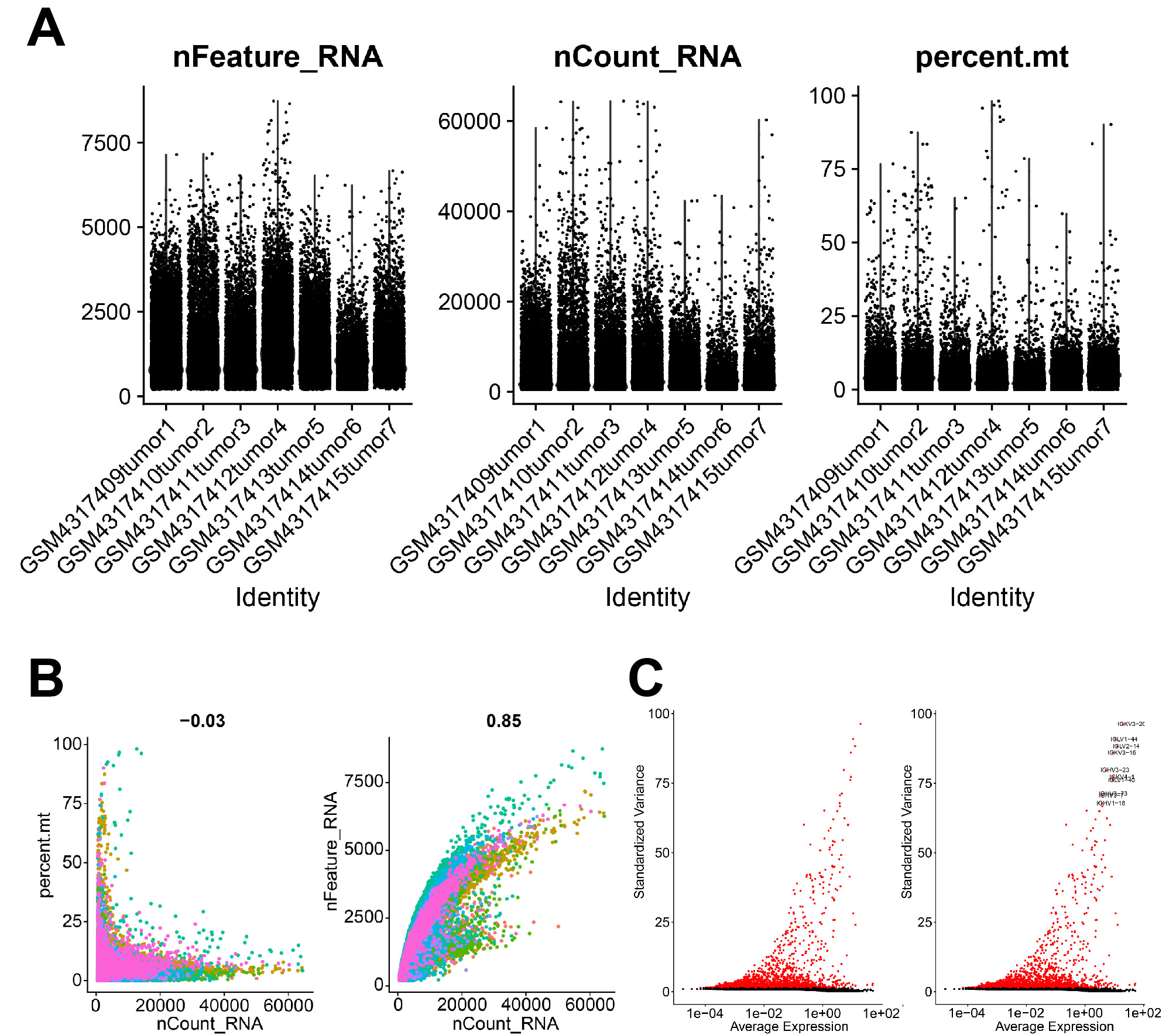

Supplement: Supplementary Figure 1 — scRNA-seq analysis of DEGs in esophageal cancer tissue samples. (A), Quality control for the 69756 cells in cancer tissue samples from seven esophageal cancer patients. Three scatter plots respectively represent the number of nFeature_RNA, nCount_RNA and percent.mt in each cell. (B), The correlation between nCount and percent.mt (left), and the correlation between nCount and nFeature (right). (C), Highly variable genes among 21240 genes screened by analysis of variance. Red dots represent highly variable genes and black dots represent invariable genes. [file Image1.jpeg]

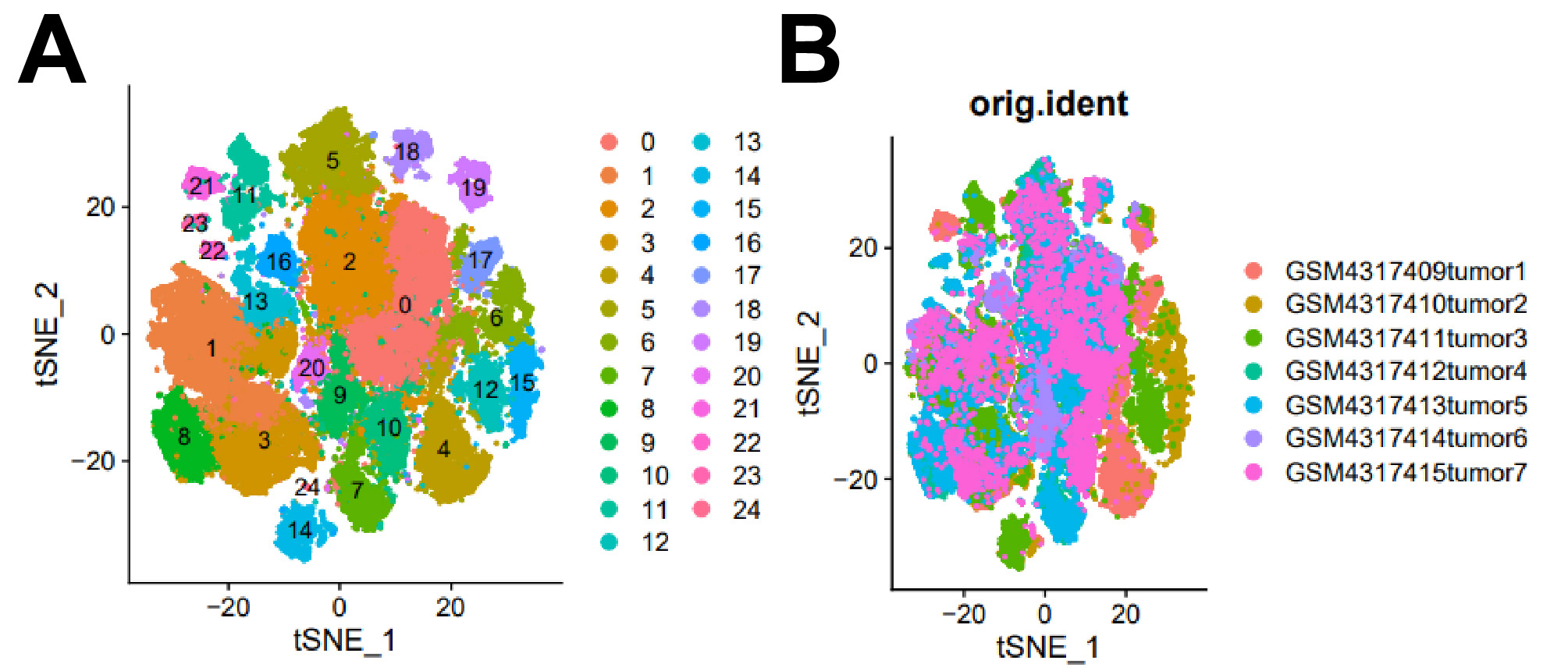

Supplement: Supplementary Figure 2 — tSNE clustering analysis was conducted on the single-cell RNA-seq data of esophageal cancer tissue samples. (A), tSNE cluster analysis clusters cells into 25 cell clusters. (B), Sample source of each cell cluster of tSNE cluster. [file Image2.jpeg]
