# Supplementary material for: M2 macrophage infiltration drives tumor progression and identifies a multigene prognostic signature in esophageal cancer
Source: Front Immunol. 2026 Feb 2;16:1659048. doi: 10.3389/fimmu.2025.1659048 (PMC12907416; doi:10.3389/fimmu.2025.1659048)
Supplement: Supplementary file 3 [file Table1.docx]

**Table S1.** RT-qPCR primer sequence

| **Gene** | **Sequence** |
| --- | --- |
| IL1RN | F: 5’- CCCCATGGCTTTAGAGACGA -3’ |
|  | R: 5’- GAGCATGAGGCTCAATGGGT -3’ |
| IL36G | F: 5’- ATCAATCAATCACTGTTGCTGTT -3’ |
|  | R: 5’- TAGCTGCAATGTGGGCTGTT -3’ |
| GAPDH | F: 5’- AATGGGCAGCCGTTAGGAAA -3’ |
|  | R: 5’- GCGCCCAATACGACCAAATC -3’ |
