# Supplementary material for: M2 macrophage infiltration drives tumor progression and identifies a multigene prognostic signature in esophageal cancer
Source: Front Immunol. 2026 Feb 2;16:1659048. doi: 10.3389/fimmu.2025.1659048 (PMC12907416; doi:10.3389/fimmu.2025.1659048)
Supplement: Supplementary file 4 [file Table2.docx]

**Table S2** Number of cells in each cell cluster in tSNE cluster analysis

| **Cell type** | **Number** |
| --- | --- |
| Macrophage | 16540 |
| Treg cell | 12900 |
| NK cell | 14739 |
| B cell | 5636 |
| Exhaustion CD4+ T cell | 3343 |
| Cytotoxic T cell | 2337 |
| Mast cell | 1029 |
| Others | 1048 |
| Sum | 57572 |
