# Supplementary material for: M2 macrophage infiltration drives tumor progression and identifies a multigene prognostic signature in esophageal cancer
Source: Front Immunol. 2026 Feb 2;16:1659048. doi: 10.3389/fimmu.2025.1659048 (PMC12907416; doi:10.3389/fimmu.2025.1659048)
Supplement: Supplementary file 5 [file Table3.docx]

**Table S3. Clinical and Model-Based Prognostic Factors Associated with Overall Survival in Patients.**

| Characteristics | Total(N) | Univariate analysis | | Multivariate analysis | |
| --- | --- | --- | --- | --- | --- |
|  |  | Hazard ratio (95% CI) | P value | Hazard ratio (95% CI) | P value |
| Age | 163 |  |  |  |  |
| <= 60 | 83 | Reference |  |  |  |
| > 60 | 80 | 0.858 (0.525 - 1.402) | 0.541 |  |  |
| Pathologic T stage | 145 |  |  |  |  |
| T1 | 27 | Reference |  |  |  |
| T2 | 37 | 0.868 (0.388 - 1.940) | 0.729 |  |  |
| T3&T4 | 81 | 1.211 (0.599 - 2.450) | 0.594 |  |  |
| Pathologic N stage | 144 |  |  |  |  |
| N0 | 66 | Reference |  | Reference |  |
| N1 | 63 | 2.853 (1.515 - 5.373) | **0.001** | 3.125 (1.477 - 6.615) | **0.003** |
| N2&N3 | 15 | 3.602 (1.483 - 8.745) | **0.005** | 4.595 (1.660 - 12.721) | **0.003** |
| Pathologic M stage | 129 |  |  |  |  |
| M0 | 121 | Reference |  | Reference |  |
| M1 | 8 | 5.075 (2.312 - 11.136) | **< 0.001** | 3.588 (1.547 - 8.324) | **0.003** |
| Risk Model | 163 |  |  |  |  |
| Low | 81 | Reference |  | Reference |  |
| High | 82 | 1.591 (1.253 - 2.607) | **0.036** | 1.759 (1.021 - 3.289) | **0.017** |
